# Docetaxel weekly regimen in conjunction with RF hyperthermia for pretreated locally advanced non-small cell lung cancer: a preliminary study

**DOI:** 10.1186/1471-2407-7-189

**Published:** 2007-10-06

**Authors:** Zhu Jiang, Wang Yan, Jiang Ming, Yang Yu

**Affiliations:** 1 Cancer Center of West China (Hua Xi) Hospital, Si Chuan University, Guo Xue Street, City of Cheng Du, Si Chuan, 610041, China; 2 West China 2nd University Hospital, Si Chuan University, Ren Min Nan Road, City of Cheng Du, Si Chuan, 610041, China

## Abstract

**Background:**

To evaluate the feasibility and therapeutic effect of chemotherapy combined with regional radio frequency hyperthermia for pretreated locally advanced non-small cell lung cancer.

**Methods:**

29 patients with stage III non-small cell lung cancer were enrolled in present study, received chemotherapy up to 4 cycles and radio frequency hyperthermia up to 32 times. The primary end points were grade 3,4 hematological or non-hematological toxicities and progression free survival, the secondary end points were response rate, tumor control rate and overall survival. Method of Kaplan-Meier was used for the survival analysis.

**Results:**

21 patients completed their arranged treatments. The most common grade 3,4 toxicity was neutropenia (24.1%). Median progression free survival was 4 months (range 0–13 months), one year progression free survival rate was 10.3%. Overall response rate was 25.9%, tumor control rate was 66.6%. Median overall survival was 11 months (range 2–18^+ ^months), one year overall survival rate was 44.8%.

**Conclusion:**

Treatment of chemotherapy in conjunction with regional hyperthermia was safe and well tolerant, it suggested an impressive tumor control rate and an acceptable one year progression free survival. Further study might be needed.

## Background

Non-small cell lung cancer accounts for at least 80% of all lung neoplasma, and about 35% of these patients present with locally advanced unresectable disease [1]. Systemic chemotherapy with the platinum-containing regimens plus concurrent regional radiation is generally accepted as the standard first-line treatment for inoperable locally advanced non-small cell lung cancer, meta-analysis confirmed the response and survival benefits, but the large number of patients will experience disease progression or tumor relapse [2-6]. For patients with a good performance status, second-line chemotherapy is recommended. Several third-generation chemotherapeutic agents had been evaluated in the second line setting, docetaxel maybe the most extensively studied one [7-10]. Recent studies indicated that second-line chemotherapy with docetaxel weekly as a single-agent regimen for pretreated non-small cell lung cancer was well tolerant and had an acceptable response rate and a reasonable median survival time about 26.1 weeks [7,11,12]. Obviously, neither physicians nor patients are satisfied with this.

Empirical studies indicated that heat played a lethal effect to human tumor cell lines, and heat enhanced cytotoxic effect of chemotherapeutics [13-15]. Along with the development of technology, hyperthermia improves therapeutic effect of superficial tumors, soft tissue sarcoma, osteosarcoma, prostate cancer, breast cancer and some recurrent or advanced solid tumors [16-21]. There are rare reports that demonstrate the advantage of adding hyperthermia to chemotherapy as the second-line therapy for pretreated locally advanced non-small cell lung cancer. In present study, we combined docetaxel weekly chemotherapy with external regional radio frequency (RF) hyperthermia as the second-line treatment for patients with inoperable stage III non-small cell lung cancer who had failed to response to the prior platinum-containing chemotherapy. The toxicities and progression free survive (PFS) were primarily investigated.

## Methods

### Patients selection

Eligible patients must had histological or cytological proof to be inoperable stage IIIa or IIIb non-small cell lung cancer, and should have at least one measurable lesion. They experienced only one platinum-containing regimen as the first-line treatment, whether accompanied with irradiation or not. They failed to respond to the first-line treatment, and proved to be still inoperable by spiral computed tomograph. In addition, patients should be with a ECOG performance status ≤ 2, and have normal hematological index (neutrophil count ≥ 2 × 10^9^/l, platelet count ≥ 100 × 10^9^/l, haemoglobin ≥ 100 g/l) and acceptable biochemical analysis result (normal bilirubin level, alanine aminotransferase ≤ 2.5 × upper limit of normal (ULN) and serum creatinine level ≤ 1.5 × ULN). Patients with metal foreign matter, fever (≥ 38°C), significant hemorrhagic tendency, and seriously impaired function of lung or heart were excluded. Every patient enrolled in this study had signed a written informed consent form approved by Si Chuan University Hua Xi Medical Ethics Committee, which also approved the study protocol.

### Chemotherapy regimen

A regimen of docetaxel 40 mg/m^2 ^intravenous infusion on day1, 8 and 15, repeated every 4 weeks was chosen. Docetaxel was administered intravenously within 1 hour. Prophylactic oral corticosteroid premedication (dexamethasone 8 mg) was given 3 times over 2 days. Target sum of chemotherapy would be 4 cycles.

Dose adjustment should be done when grade 4 hematological or grade 3,4 non-hematological toxicities appeared, a dose reduction was of 25% in subsequent cycles. Docetaxel infusion could not be delayed for over 2 weeks since patient discontinuation.

### Hyperthermia regimen

We used a radio frequency external heat system (Ho Kai company, China) working frequency was 13.8 MHz to perform regional hyperthermia. Primary lesion was chosen to be the target area, covered by an applicator 20 cm in diameter. Hyperthermia was administered 1 hour after chemotherapeutics were given, the total heated time was up to 60 minutes, twice a week, aimed to 32 times, interval between 2 procedures should exceed 48 hours. Target temperature ranged from 41°C to 43°C, we instructed every patient to mention any abnormal feelings, so that we could adjust settings such as power output, or place a water bolus to ease the uncomfortable.

### Evaluation of response and toxicity

The response had been estimated within a week after every 2 cycles of chemotherapy were given, and to be confirmed in 4 weeks of responding patients. We used contrast-enhanced spiral computed tomograph to measure the largest diameter of target tumor, response criteria was based on Response Evaluation Criteria In Solid Tumors (RECIST)[22]. Toxicity evaluation was done in all patients according to NCI CTC version 2.0 [23].

### Follow up

Eligible patients were visited once a month after treatment starting, their PFS and overall survival (OS) time was recorded. Disease evaluation was performed every 3 months or when necessary. The time limit of follow-up was 18 months in present study.

### Statistical analysis

Main objective of present study was to evaluate the safety and therapeutic effect of docetaxel weekly chemotherapy combined with RF hyperthermia for pretreated locally advanced non-small cell lung cancer. The primary end points were grade 3,4 hematological or non-hematological toxicities and PFS, the secondary end points were response rate, tumor control rate and OS.

Kaplan-Meier survival curve was used to describe PFS and OS. Statistical work was performed by soft ware SPSS 13.0 (SPSS Inc. USA). Lost patients were recorded as death, their PFS were calculated up to the last visit as well as OS, except they had already experienced tumor progression.

## Results

### Patients' characteristics

From June 2005 to December 2005, 29 pretreated patients with stage III non-small cell lung cancer were included in this study, 8 patients in stage IIIb accompanied with pleural effusion, who were not suitable for radiation. And other 10 patients refused to sign the informed consent form for radiotherapy. Overview of this study was fully explained to every eligible patient, every enrolled patient had signed an informed consent form and asked to keep one copy. Patients' characteristics were presented in Table 1.

**Table 1:** Patients' characteristics

Characteristics	Number of patients	Percentage
Patients treated	29	-
Age		
Median	57	-
Range	35–72	-
Gender		
Male	21	72.4
Female	8	27.6
Histological subtype		
Squamous carcinoma	11	37.9
Adenocarcinoma	18	62.1
Stage		
IIIa (T_2_N_2_M_0_/T_3_N_2_M_0_)	12 (8/4)	41.4
IIIb (T_2_N_3_M_0_/T_4_N_1_M_0_/T_4_N_2_M_0_)	17 (2/8/7)	58.6
ECOG performance status		
0	12	41.4
1	12	41.4
2	5	17.2
Prior chemotherapy (cycles)		
Gemcitabine and cisplatin	14 (46)	48.3%
Vinorelbine and cisplatin	8 (21)	27.6%
Paclitaxel and cisplatin	7 (26)	24.1%
Prior radiotherapy	11	37.9%

### Treatment exposure

249 docetaxel infusions and 626 times of hyperthermia were administered totally. Median dosage of docetaxel was 40 mg/m^2 ^(range, 30–40 mg/m^2^), median endurable temperature was 42.3°C (range, 41.3–43.0°C). There were 13 patients (44.8%) completing 4 cycles of chemotherapy and 32 times of hyperthermia, 2 patients (6.9%) had been administered only 1 cycle of chemotherapy and 2 times of hyperthermia.

### Toxicity

8 patients (27.6%) discontinued study treatment, 6 of them due to toxicities: 4 because of grade 3 neutropenia, and 2 because of grade 4 neutropenia; the other 2 patients withdrew their informed consent form. No patient terminated treatment because of the side effect of RF hyperthermia.

6 patients (20.7%) experienced a dose reduction to 30 mg/m^2 ^due to toxicities (5 patients due to neutropenia, 1 patient due to arthralgia).

Treatment delays were applied in 5 patients (17.2%), 4 delays occurred due to neutropenia, the other one occurred due to abnormal level of alanine aminotransferase (>2.5 × ULN).

3 patients (10.3%) experienced grade 1 empyrosis after heated, no related treatment was administered. This side effect released in 48 hours, not even one time of hyperthermia was delayed for this reason.

Main toxicities were summarized in Table 2.

**Table 2:** Main toxicities per patient

Toxicity	Grade 1	Grade 2	Grade 3	Grade 4
Hematological				
Hemoglobin	2(6.9%)	0	0	0
Neutrophils	5(17.2%)	4(13.8%)	5(17.2%)	2(6.9%)
Platelets	1(3.4%)	1(3.4%)	0	0
Non-hematological				
Palpitation	1(3.4%)	-	-	-
Edema	2(6.9%)	1(3.4%)	0	0
Fatigue	6(20.7%)	2(6.9%)	0	0
Sweating	4(13.8%)	0	-	-
Weight loss	3(10.3%)	1(3.4%)	0	-
Alopecia	5(17.2%)	2(6.9%)	-	-
Nausea	5(17.2%)	2(6.9%)	1(3.4%)	-
Taste disturbance	1(3.4%)	0	-	-
Vomiting	2(6.9%)	3(10.3%)	0	0
SGOT (AST)	3(10.3%)	0	1(3.4%)	0
SGPT (ALT)	1(3.4%)	1(3.4%)	1(3.4%)	0
Insomnia	3(10.3%)	0	0	-
Arthralgia	2(6.9%)	0	1(3.4%)	0
Headache	2(6.9%)	0	0	0
Myalgia	0	0	1(3.4%)	0

### Response

There were 27 patients (93.1%) who had been evaluated for response. No patient achieved a complete remission (CR), 7 patients (25.9%) achieved a partial remission (PR), 11 patients (40.7%) were evaluated as a stable disease (SD), and 9 patients (33.3%) experienced a progression disease (PD). Overall response rate was 25.9%, tumor control rate (CR + PR + SD) was 66.6%.

### Survival analysis

**Figure 1:**
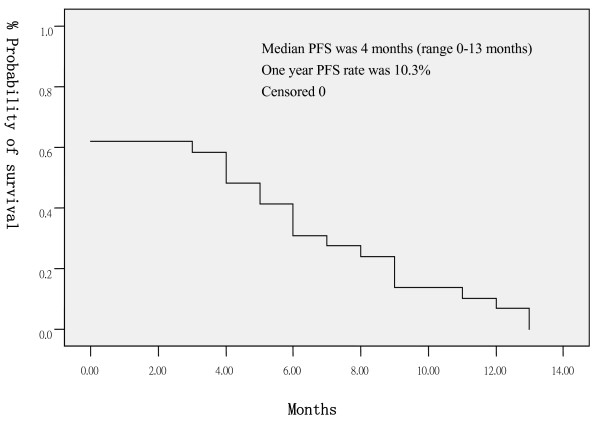
Progression free survival.

**Figure 2:**
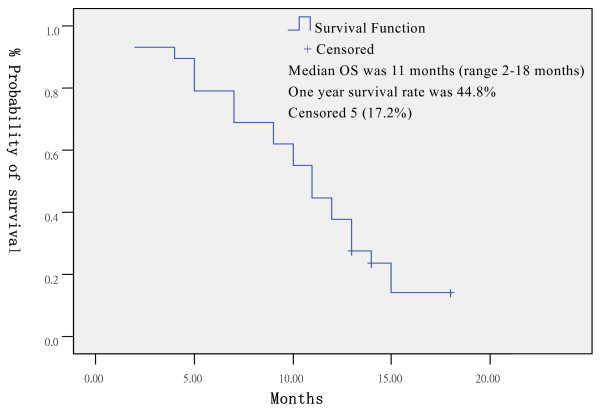
Overall survival.

The median follow-up time was 16 months (range 12–18 months), 4 patients (13.8%) were lost at follow up, their PFS/OS (months) was recorded as following: 0/2, 0/2, 0/5, 0/7. Median PFS was 4 months (range 0–13 months, 95% CI: 1.9–6.1 months), one year progression free survival rate was 10.3% (Figure 1). Median OS was 11 months (range 2–18^+ ^months, 95% CI: 9.3–12.8 months), one year survival rate was 44.8% (Figure 2). At the time of analysis, 5 patients were still alive.

## Discussion

Docetaxel is recommended for second-line chemotherapy of non-small cell lung cancer, and weekly administration regimen showed a good safety, but recent studies resulted that the response rate and survive was still poor [12]. There are rare studies about hyperthermia for lung cancer, even it had been reported to improve the therapeutic efficacy in some drug insensitive tumors such as prostate cancer and soft tissue sarcoma [17,18,20].

Structure of vascular in malignant tissue is great different from normal tissue, it was reported that the blood flow rate in normal tissue would be 30 times greater than tumor tissue [24]. This difference makes temperature of tumor tissue significantly higher than normal tissue under the same heat condition. Cytotoxic effect of several antitumor agents include docetaxel can be enhanced by raising temperature [25]. Hyperthermia also shows as a complementation of radiotherapy [26]. RF hyperthermia is powered by electromagnetic wave of a certain wavelength, it has the ability to go through whole human body and transforms to heat when different mediator encountered. Current study was designed to evaluate feasibility and efficacy of docetaxel plus RF hyperthermia for patients failed to response to prior platinum-containing chemotherapy.

A total of 29 eligible patients enrolled in present study, 12 patients were of stage IIIa, 17 patients were of stage IIIb. 21 patients (72.4%) completed the arranged study treatment, while 8 patients (27.6%) discontinued. Main hematological toxicity was neutropenia, 7 patients (24.1%) experienced Grade 3,4 neutropenia, most of them were grade 3. With G-CSF hypodermic injection, this toxicity was well controlled. None of grade 4 non-hematological toxicities was observed, but grade 3 nausea, transaminase increasing, arthralgia and myalgia had been observed. Only 3 patients experienced grade 1 empyrosis, and they all self-recovered. Results above showed the comparable toxicities[7,11], it might indicate that regional RF hyperthermia does not increase the toxicity of chemotherapy.

The therapeutic efficacy of the study treatment was acceptable, a response rate of 25.9% and a tumor control rate of 66.6% were better than docetaxel alone. A median PFS of 4 months and a median OS of 11 months might not be satisfied, but compared with docetaxel alone (median OS ranged from 6.4 to 9.2 months), these results were encouraging [7,8].

## Conclusion

Docetaxel weekly regimen in conjunction with regional RF hyperthermia acted as the second-line treatment for pretreated locally advanced non-small cell lung cancer was safe and well tolerant. The results of current study also suggested advantages in response and survival. A further phase III study is supported by these results.

## Competing interests

The author(s) declare that they have no competing interests.

## Authors' contributions

ZJ designed this study and was in charge of the clinical protocol. WY analyzed the data and drafted the manuscript. ZJ, JM and YY enrolled patients in the clinical protocol. All authors read and approved the final manuscript.

## Pre-publication history

The pre-publication history for this paper can be accessed here:


